# News and Miscellany

**Published:** 1900-09

**Authors:** 


### News and Miscellany.

---

**Dr. W. N. Rogers,** of Waco, died at his home in that city May 29, 1900.

---

**Wanted.**—Two copies of the "Red Back" for February, 1900. Two months' subscription, for each copy, will be credited to any subscriber who will oblige us by sending them.

---

**For Sale:**—$1600 practice, nice home and office, in country village, Central Texas, no opposition, people all Americans. Terms, $500, $300 cash, balance to suit. If you mean business write for particulars. Address Dr. L. C. B——

Care Texas Medical Journal.

---

**New Orleans Polyclinic.**—Physicians will find the Polyclinic an excellent means for posting themselves upon modern progress in all branches of medicine and surgery. The specialties are fully taught, particularly laboratory work. Fourteenth annual session opens November 12, 1900. For further information address Dr. Isadore Dyer, Secretary, New Orleans Polyclinic, New Orleans.

---

**The Viskolein Company,** whose advertisement please see, calls our attention to the fact that we used in our August issue an electro of Viskolein which had been made when Viskolein was in the hands of Maltbie Chemical Co., and before Viskolein Chemical Co. took hold of it. As the use of the wrong electro may have misled some of our readers we make this statement, and call attention to the advertisement. Viskolein is pronounced an ideal treatment for typhoid fever, and is an ideal all round intestinal antiseptic.

---

**Pasteur Institute in Baltimore.**—"The Pasteur Department of the Baltimore City Hospital," has been founded and is controlled by the College of Physicians and Surgeons of that city. Prof. Thos. S. Latimer, M. D., is Physician in Chief; Prof. N. G. Keirle, A. M., M. D., is Chief of Laboratory, with Prof. Julius Friedenwald, A. B., M. D., is his Assistant Chief of Laboratory. Without the Pasteur preventive treatment, the mortality from bites of rabid animals is 16 to 25 per cent. Of those treated by the Pasteur method, the mortality is about one-thirteenth of 1 per cent.

### To Mask Quinine.

---

℞ Quinæ sulphat .................... 4

Acidi citrici .......................10

Syr. simp. .........................10

Syr. aurantii flor..................10

Aq. destillat, ad................20 c. c.

M. Sig.: Ten drops in 50 grams of water. Add 3 grams of sodium bicarbonate and drink during effervescence.—Ann. de Med. et Chir. Inf.—*Ex.*

---

According to the *Journal of the American Medical Association*, the city of Sassari, on the island of Sardinia, has been completely freed from mosquitoes. A chart was made showing every discoverable breeding place for mosquitoes, and these were all treated twice a month with coal oil. It is said that the entire cost for a city of 50,000 inhabitants should be about $250, or $5 per 1000. Cheap enough.

